# Dimerization reactions of aryl selenophen-2-yl-substituted thiocarbonyl *S*-methanides as diradical processes: a computational study

**DOI:** 10.3762/bjoc.13.44

**Published:** 2017-03-03

**Authors:** Michael L McKee, Grzegorz Mlostoń, Katarzyna Urbaniak, Heinz Heimgartner

**Affiliations:** 1 Auburn University, Department of Chemistry and Biochemistry, Auburn, AL, 36849, USA; 2 Department of Organic and Applied Chemistry, University of Łódź, Tamka 12, PL 91-403 Łódź, Poland; 3 Department of Chemistry, University of Zurich, Winterthurerstrasse 190, CH-8057 Zurich, Switzerland

**Keywords:** 1,3-dipolar cycloadditions, reaction mechanisms, reactive intermediates, thiocarbonyl *S*-methanides, thioketones

## Abstract

An intriguing stepwise diradical mechanism of the dimerization of the reactive intermediate (thiocarbonyl *S*-methanide) appearing in the reaction of phenyl selenophen-2-yl thioketone with diazomethane was studied by means of computational methods. The preferred formation of the unusual macroheterocycle, competitive with the 1,3-ring closure leading to a thiirane and the head-to-head dimerization yielding a 1,4-dithiane derivative, respectively, was explained based on the analysis of the structure of the favored conformer of the intermediate, delocalized diradical species. The influence of selenium as a ‘heavy atom’ for stabilization of this intermediate has been emphasized.

## Introduction

Thiocarbonyl *S*-methanides **1** belong to the class of *S*-centered 1,3-dipolar species, which were identified by Huisgen as reactive intermediates formed via nitrogen extrusion from 2,5-dihydro-1,3,4-thiadiazoles **2** [1–2]. In spite of the fact that several methods are reported for the preparation of these precursors, the most convenient access comprises the treatment of a C=S substrate with diazomethane or its derivatives. Depending on the substitution pattern, 2,5-dihydro-1,3,4-thiadiazoles **2** display different thermal stability. Whereas sterically bulky aliphatic substituents increase the temperature required for the decomposition, 2,2-diaryl-substituted derivatives can be generated only at low temperature, typically at −60 °C in THF solution.

**Scheme 1:**
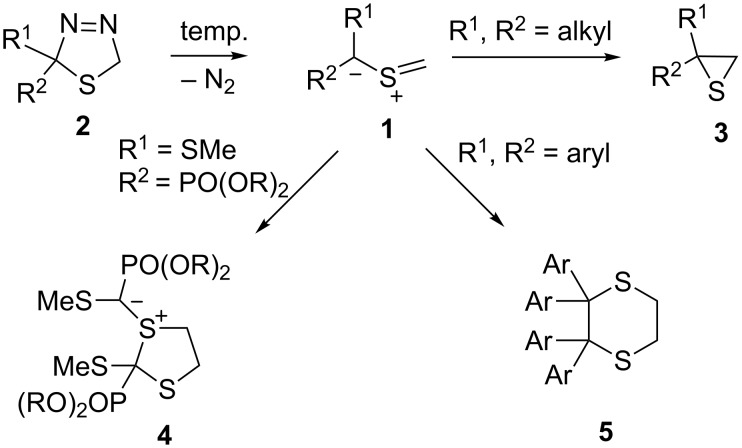
Generation and typical reactions of the reactive dialkyl and diaryl thiocarbonyl *S*-methanides **1**.

The in situ generated thiocarbonyl *S*-methanides **1** are considered as electron-rich 1,3-dipoles, which easily react with electron-deficient dipolarophiles, yielding a plethora of five-membered heterocycles [2]. In addition, some cycloaliphatic thiocarbonyl *S*-methanides were shown to react with strongly electron-deficient cyano-substituted ethenes via zwitterionic intermediates to yield also seven-membered cyclic ketene imines, which easily undergo further conversions [3–5]. In the case of similar thiocarbonyl *S*-isopropanides, the intermediate zwitterions, formed in the course of the attempted [3 + 2]-cycloaddition with electron-deficient ethenes, undergo 1,3-electrocyclization yielding mixtures of stereoisomeric cyclopropanes in addition to the expected 5-membered thiolanes (tetrahydrothiophenes) [6]. In the absence of a suitable dipolarophile, thiocarbonyl *S*-methanides **1** undergo either 1,3-dipolar electrocyclizations to give the isomeric thiiranes **3** or dimerize leading to five- or six-membered *S*-heterocycles (Scheme 1) [1–2].

The type of the dimeric product depends on the substituents located at the terminal C-atoms of the 1,3-dipole. In the case of polar groups, such as sulfonyl or phosphonyl moieties, the dimerization leads to five-membered 1,3-dithiolane derivatives **4** (sulfoniumylides) [7–8]. Diaryl-substituted thiocarbonyl *S*-methanides **1** react in the absence of a dipolarophile, with no exception, via a head-to-head dimerization yielding sterically crowded 1,4-dithianes of type **5** [9–10]. The analogous head-to-head dimerization course was reported for the thiocarbonyl *S*-methanide derived from benzyl octafluorodithiopentanoate [11].

**Figure 1:**
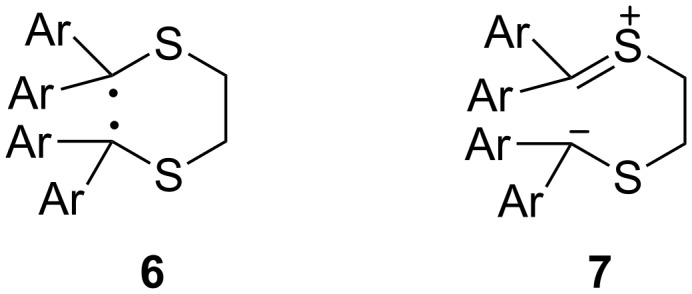
Structures of the reactive intermediates as a diradical **6** or a zwitterion **7** in the course of the dimerization of a reactive diaryl thiocarbonyl *S*-methanides.

The reaction mechanisms of the dimerization processes have not been studied in detail yet. Whereas the formation of the 1,3-dithiolane **4** can be explained via a concerted [2 + 3] cycloaddition of **1** as a 1,3-dipole with the activated C=S bond of **1**, the dimerization leading to **5** seems to occur stepwise via an intermediate stabilized 1,6-diradical **6**. In an earlier report, however, a zwitterionic intermediate **7** has been proposed as an intermediate (Figure 1) [10].

Later, the mechanism of this dimerization was studied by means of computational methods, and the intermediacy of diradical **6** was formulated based on B3LYP calculations [12] and a concerted pathway was excluded.

## Results and Discussion

**Scheme 2:**
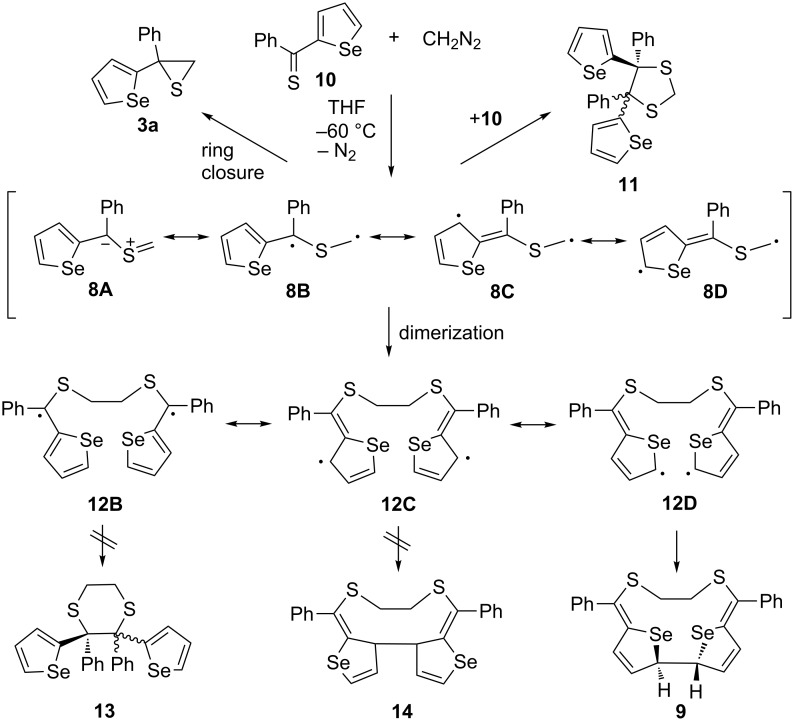
The in situ generation of phenyl selenophen-2-yl *S*-methanide (**8**) and its competitive reactions: 1,3-ring closure, head-to-head dimerization, and macroheterocycle **9** formation via an intermediate diradical species **8B**–**D**.

In a recent publication, reactions of hetaryl phenyl and dihetaryl thioketones with diazomethane have been described, and the formation of 1,3-dithiolanes as well as unexpected, hitherto unknown, dimers of the intermediate thiocarbonyl *S*-methanide was observed already at −70 °C. The structure of the dimer of phenyl selenophen-2-yl thiocarbonyl *S*-methanide (**8**) was determined as the unusual macroheterocycle **9** [13] (Scheme 2). Further, the study showed that the presence of the selenophenyl substituent is essential for this type of dimerization. In addition, substituents located in 4-position of the phenyl ring influence the yield of **9** formed in a competitive reaction with the formal [2 + 3]-cycloaddition of **8** with the starting thioketone **10** leading to the sterically crowded 1,3-dithiolane **11**.

The structure of the dimer **9**, confirmed by an X-ray analysis [13], suggests that it is formed via the intermediate delocalized 1,7-diradical **8D**, which dimerizes to give the 1,14-diradical **12D** and subsequently undergoes the ring closure yielding product **9** identified as the *rac*-diastereoisomer.

The goal of the present study was the examination of the postulated reaction pathway via a diradical intermediate leading to the formation of the unusual macrocyclic dimer **9** by computational methods.

**Figure 2:**
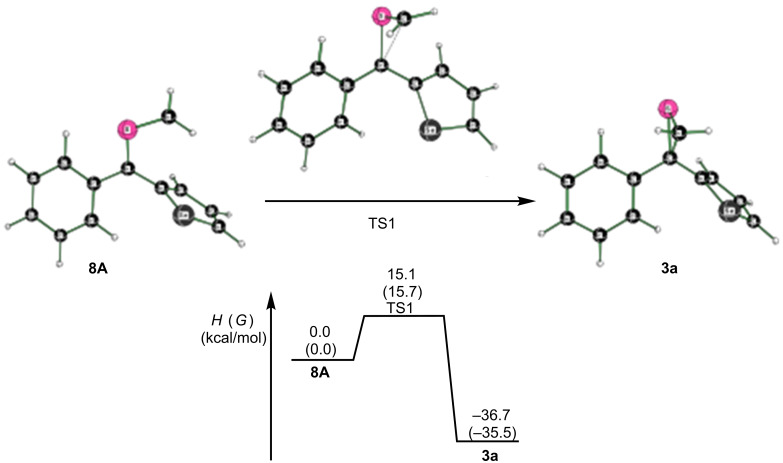
Potential 1,3-dipolar electrocyclization of thiocabonyl *S*-methanide **8A.** Computed enthalpies (free energies in parentheses) at 298 K at the SMD(tetrahydrofuran)/M06-2X/6-311+G(2df,p)//M06-2X/6-31G(d) level of theory.

First calculations were made for phenyl selenophen-2-yl thiocarbonyl *S*-methanide (**8A**), which can undergo a 1,3-dipolar electrocyclization (1,3-ring closure) to form the thiirane **3a**. This reaction has a free energy of the transition state of 15.7 kcal/mol (TS1) and is spontaneous by −35.5 kcal/mol (Figure 2).

**Figure 3:**
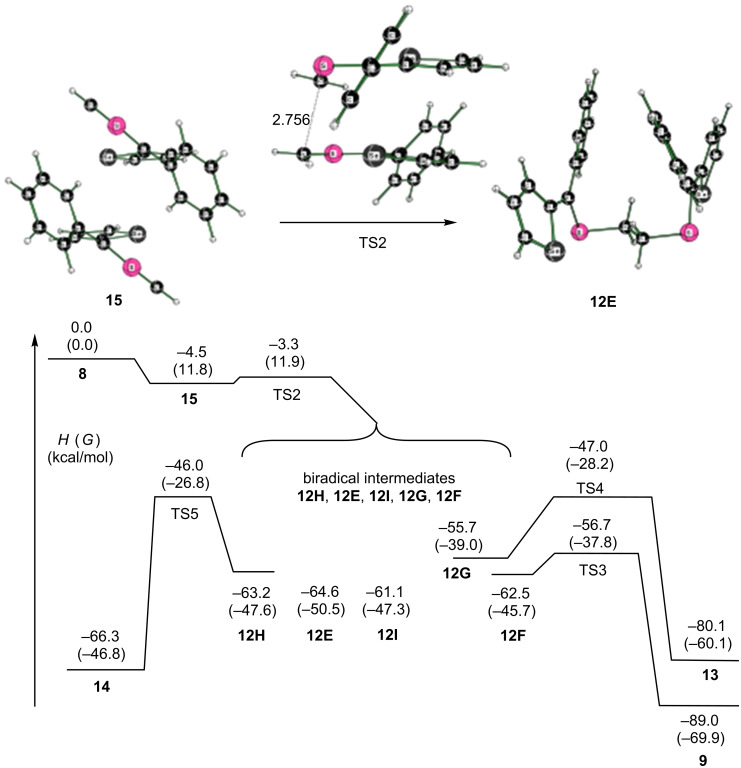
Stepwise radical dimerization of the reactive thiocarbonyl *S*-methanide **8**. Computed enthalpies (free energies in parentheses) at 298 K at the SMD(tetrahydrofuran)/M06-2X/6-311+G(2df,p)//M06-2X/6-31G(d) level of theory. The species **12E**–**I** are all biradical intermediates in the reaction mechanism and should be able to interconvert readily.

**Figure 4:**
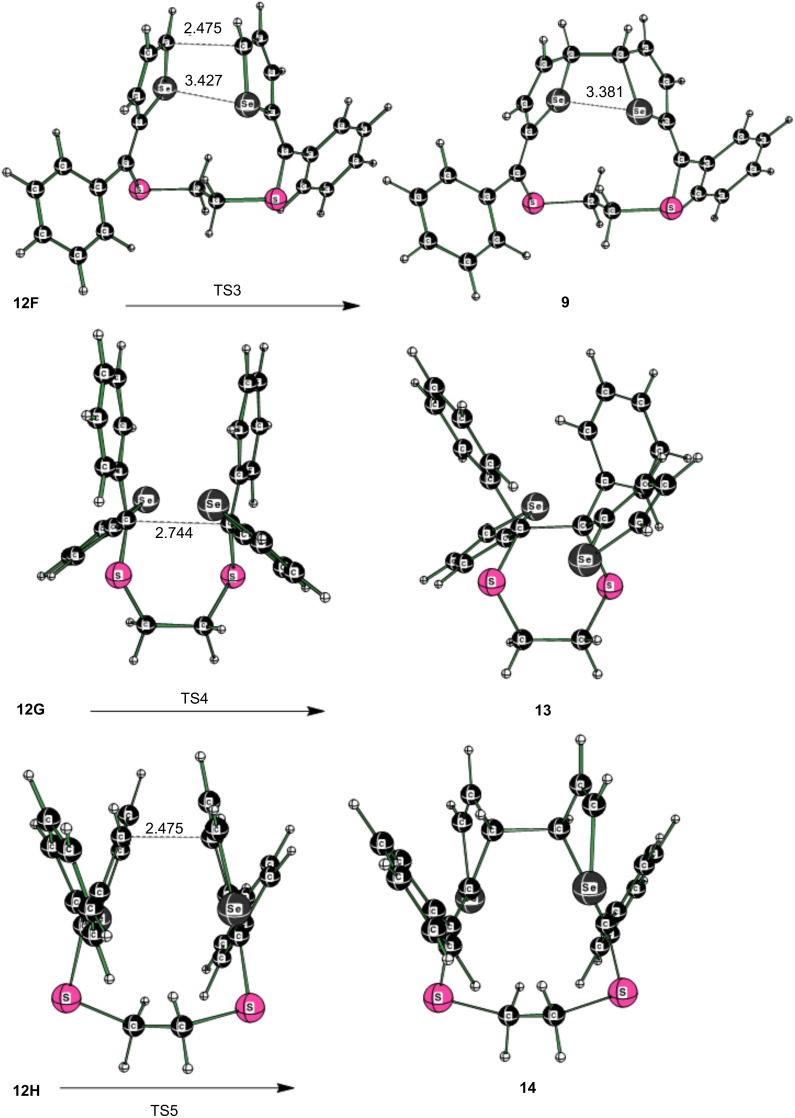
Potential competitive cyclization reactions of the intermediate diradical **12**.

In an alternative reaction, two molecules of thiocarbonyl ylide **8** initially form a complex **15**, which is bound by 4.5 kcal/mol (∆*H* (THF, 298 K)) and subsequently converts over a 1.2 kcal/mol activation barrier (TS2) forming a C–C bond to give the intermediate, delocalized diradical **12** in a spontaneous reaction (∆*G* = −50.5 kcal/mol, Figure 3). This diradical can adopt a number of conformations. While the conformer surface was not fully explored, five conformers were located (see Supporting Information File 1, Figure SI1), and three of them are considered to be able to form another C–C bond to give, potentially, products **9**, **13**, or **14** (Scheme 2, Figure 3 and Figure 4). All of the conformers **12** are diradicals as judged by a spin-squared value <S^2^> close to 1.0 (see Supporting Information File 1, Table SI1) in the broken symmetry solution.

The further conversion of the conformers of the intermediate diradical **12** into the macroheterocycle **9**, the six-membered 1,4-dithiane **13**, and the ten-membered heterocycle **14**, respectively, is governed by two factors: the unpaired spin density at the two corresponding C atoms and the strain required to reach the transition state.

**Figure 5:**
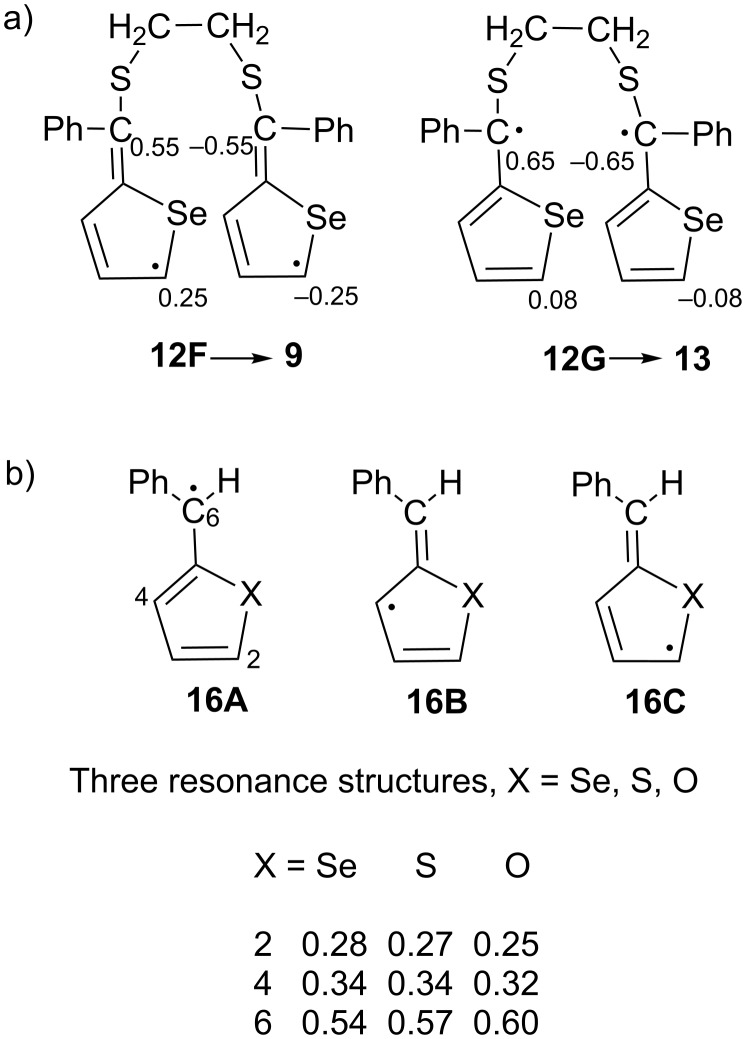
a) Spin densities in the conformers **12F** and **12G** of diradical **12**. b) Heteroatom effect on the magnitude of the spin density in the model diradical **16**.

The spin densities of the two C atoms in conformer **12F**, which is the precursor of the final product **9**, is 0.25 e^–^, which is smaller than that of the two C atoms that do not form a new C–C bond (0.55 e^−^, Figure 5). In conformer **12G**, the diradical intermediate leading to product **13**, where the two spins do couple, the spin density is 0.65 e^−^.

As a model system to determine the effect of the heteroatom on the magnitude of the unpaired spin density, calculations were made for PhC(H)C_4_XH_3_, X = Se, S, O (**16**, Figure 5). The largest spin density was found at the benzylic C(6) atom, followed by C(4), and the smallest value was calculated for C(2). However, the unpaired density on C(2) increases and the unpaired spin density on C(6) decreases for X = Se relative to X = O or S. It is likely that the less electronegative nature of Se in the five-membered ring increases the spin density on the adjacent C(2) atom. Therefore, from the perspective of spin density, the formation of the six-membered ring **13** should be preferred.

With respect to the most stable conformer **12E**, the free energy barrier of the reaction leading to **13** is 22.3 kcal/mol (**12E** → **12G** → **TS4** → **13**). The alternative pathway, which results in the formation of the new C–C bond via **12E** → **12H** → **TS5** → **14** has a higher free energy of the transition state of 23.7 kcal/mol. The lowest free energy barrier was found for the coupling of the two C(2) atoms (**12E** →**12F**→ **TS3** → **9**) with a value of 12.7 kcal/mol. These results are in accordance with the observed formation of the twelve-membered ring of dimer **9** formed in the reaction of **8** [13].

The order of the free energies of the transition states (**TS3** < **TS4** ≈ **TS5**) reflects the steric requirement to reach the transition state. For the C–C coupling in the **12F** → **TS3** reaction, the C,C distance in the reactant is 2.958 Å, which is reduced to 2.475 Å in the transition state (Figure 4). Thus, for the reaction sequence with **8**, the lowest free energy barrier is found for the initial C–C coupling leading to the diradical dimer **12**, followed by the C–C bond formation involving the C(2) centers of the selenophene rings to yield the obtained twelve-membered heterocycle **9**.

In extension of the study, calculations were performed on the related symmetrical thiobenzophenone *S*-methanide by the same computational approach (see Supporting Information File 1). The cyclization of the diradical leading to 2,2-diphenylthiirane occurs via a transition state with a free energy barrier very similar to the conversion **8** → **3a** (16.4 versus 15.7 kcal/mol, respectively). The intermediate complex of two ylide molecules converts into the 1,6-diradical with a very small enthalpic barrier (2.5 kcal/mol). The cyclization of this intermediate resulting in the formation of the six-membered 1,4-dithiane is a low energy process, which requires a free energy of only 15.3 kcal/mol. This value is significantly lower than in the studied case of the diradical intermediate **12**. These results are in good agreement with the data reported by Sustmann [12].

## Conclusion

The intriguing dimerization pathway of the phenyl selenophen-2-yl *S*-methanide **8** was elucidated by computational methods using the SMD implicit solvation model [14]. The initial step of this reaction leading to the intermediate delocalized diradical occurs via a transition state with very low activation energy. On the other hand, the calculated spin densities suggest the preference of the formation of the six-membered ring of the 1,4-dithiane, and this reaction course is observed in the case of thiobenzophenone *S*-methanide **1** (Ar^1^ = Ar^2^ = Ph). On the other hand, in the hetaryl functionalized thiocarbonyl *S*-methanide **8** the lowest activation energy was found for the C–C bond formation between C(2) atoms of the selenophen-2-yl ring, leading to the twelve-membered macrocyclic product **9** isolated from the reaction mixture. Thus, the presence of the heteroatom is of crucial importance for this new type of dimerization of aryl/hetaryl thiocarbonyl *S*-methanides of type **1** and this fact reflects the importance of so called ‘heavy atom effect’ [15] in the studied system.

Stepwise, diradical mechanisms in [3 + 2]-cycloadditions have intensively been studied [16] and recently, a computational study on their appearance in reactions of substituted acetylenes with nitrile oxides has been published [17].

## Experimental

The Gaussian 09 program system was used for all calculations [18]. The M06-2X exchange/correlation functional [19] was used with the 6-31G(d) basis set for optimization. Single-point calculations were made at the M06-2X/6-311+G(2df,p) level with the SMD implicit solvation model [14] using the standard parameters for tetrahydrofuran. Zero-point and thermal corrections were combined with solvation effects (assuming ∆*H*(solv) = ∆*G*(solv)) to obtain enthalpies at 298 K in THF (Equation 1). The calculated entropy was used to determine the –*T*∆*S* term to form free energies at 298 K in THF (Equation 2).

[1]
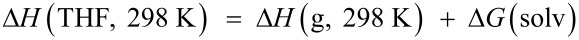


[2]
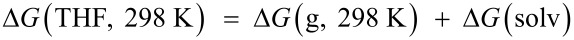


## Supporting Information

File 1 Computational data.
