# Supplementary material for: Dimerization reactions of aryl selenophen-2-yl-substituted thiocarbonyl S-methanides as diradical processes: a computational study
Source: Beilstein J Org Chem. 2017 Mar 3;13:410–6. doi: 10.3762/bjoc.13.44 (PMC5355913; doi:10.3762/bjoc.13.44)
Supplement: File 1 — Computational data. [file Beilstein_J_Org_Chem-13-410-s001.pdf]

Supporting Information

for

Dimerization reactions of aryl selenophen-2-yl-substituted thiocarbonyl *S*-methanides as diradical processes: a computational study

Michael L. McKee,*[1], Grzegorz Mlostoń*[2], Katarzyna Urbaniak[2], and Heinz Heimgartner[3]

Address: [1]Auburn University, Department of Chemistry and Biochemistry, Auburn, AL, 36849, USA, [2]Department of Organic and Applied Chemistry, University of Łódź, Tamka 12, PL 91-403 Łódź, Poland and [3]Department of Chemistry, University of Zurich, Winterthurerstrasse 190, CH-8057 Zurich, Switzerland

Email: Michael L. McKee - mckeeml@auburn.edu; Grzegorz Mlostoń - gmloston@uni.lodz.pl

*Corresponding author

Computational data

Contents:

Figure SI1. Selected conformations of the diradical **12**.

Figure SI2. Transition states for the formation of thiirane **3a**, diradical **18**, and 1,4-dithiane **5a.**

Figure SI3. Free activation energies for the radical transformations of thiobenzophenone.

Table SI1. Zero-point energies (kcal/mol), integrated heat capacities (kcal/mol, 298 K), entropies (kcal/mol·K), total energies (hartrees) with m06-2x and with the smd solvation model, relative enthalpies (kcal/mol, 298 K) in THF, solvation free energies (kcal/mol) in THF, and relative free energies (kcal/mol, 298 K) in THF.

Table SI2. Cartesian coordinates of relevant species at the M06-2X/6-31G(d) level.

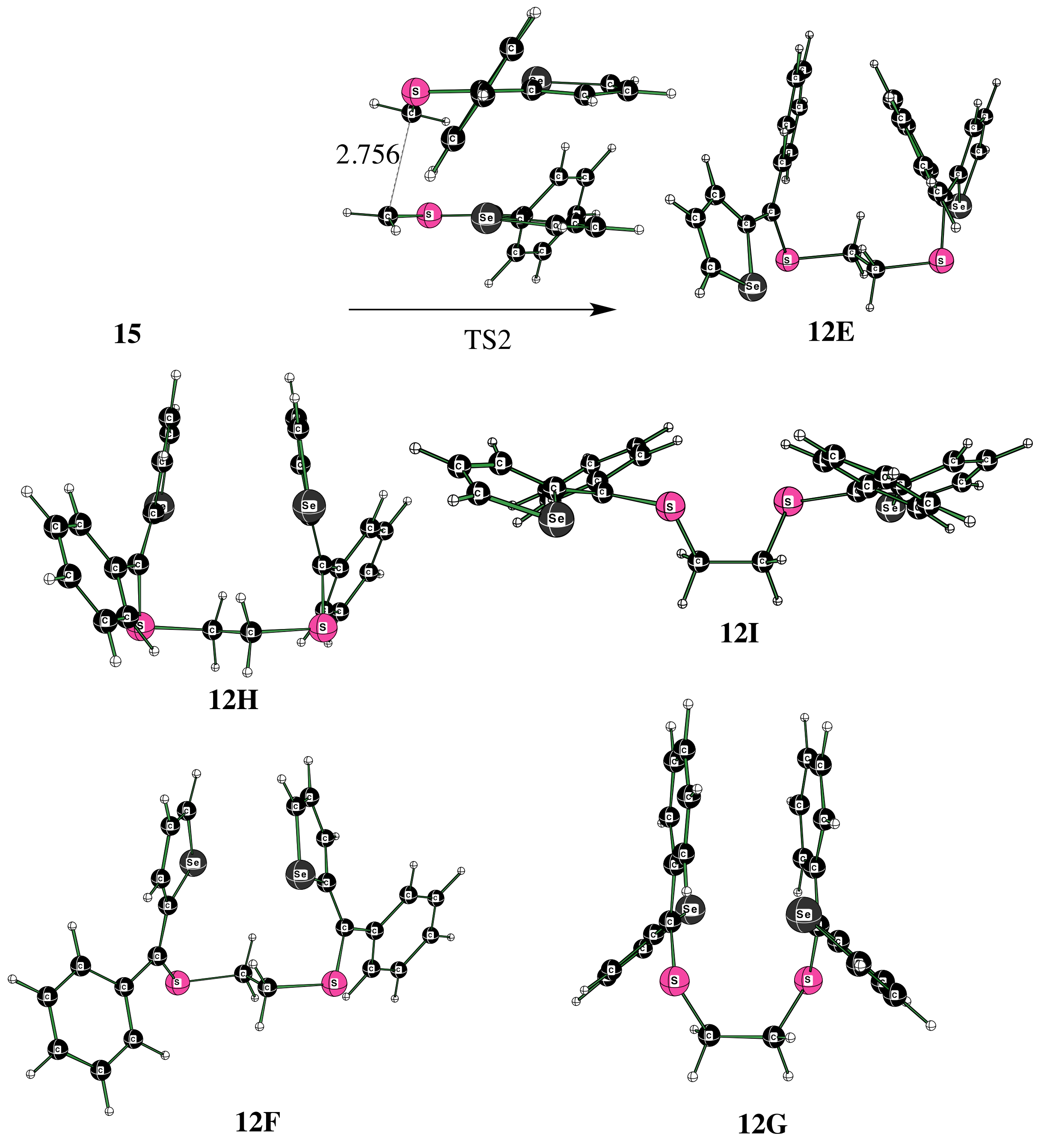

Figure SI1. Selected conformations of the diradical **12**.

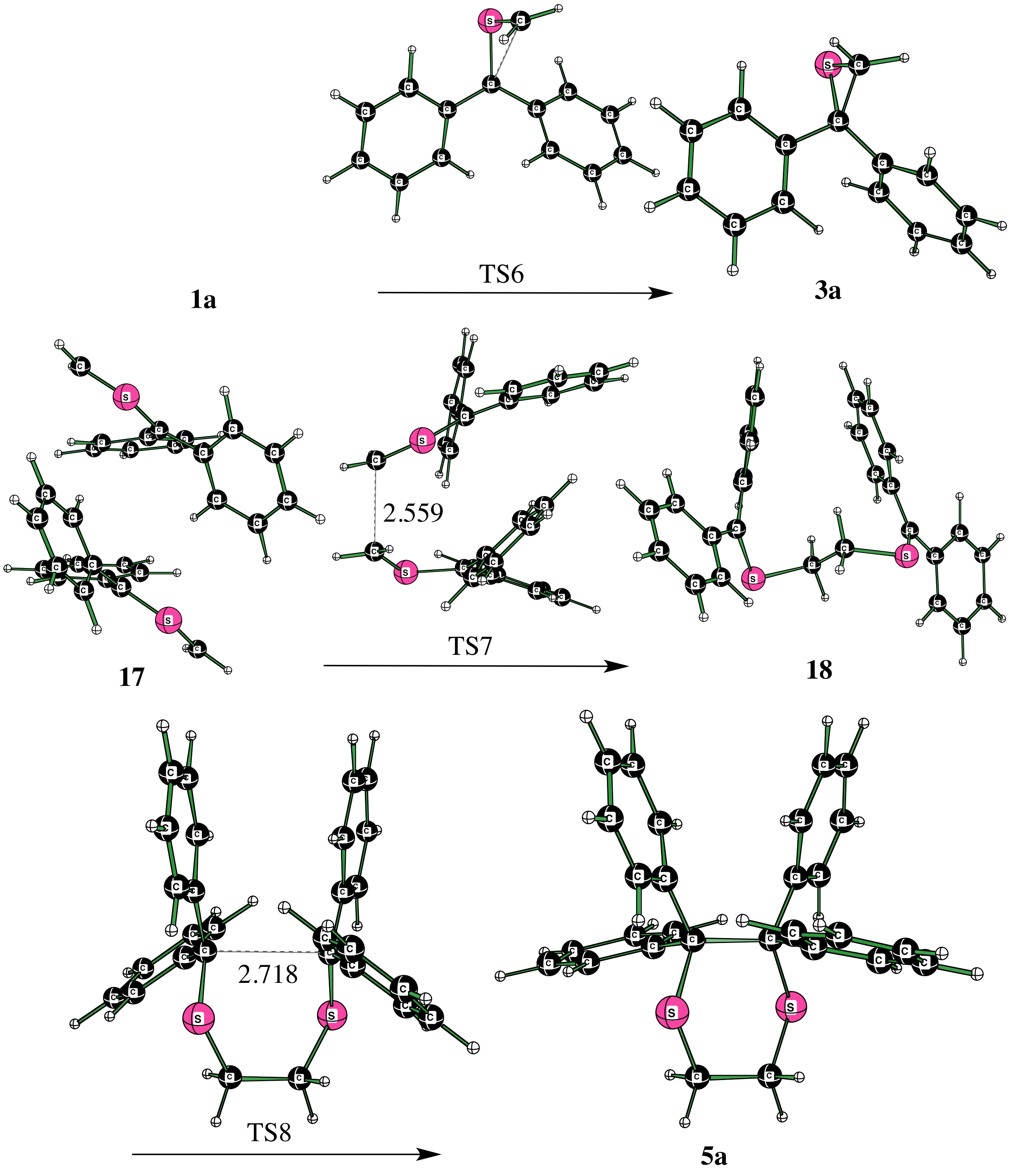

Figure SI2. Transition states for the formation of thiirane **3a**, diradical **18**, and 1,4-dithiane **5a**, respectively, in the case of thiobenzophenone *S*-methanide **1a**.

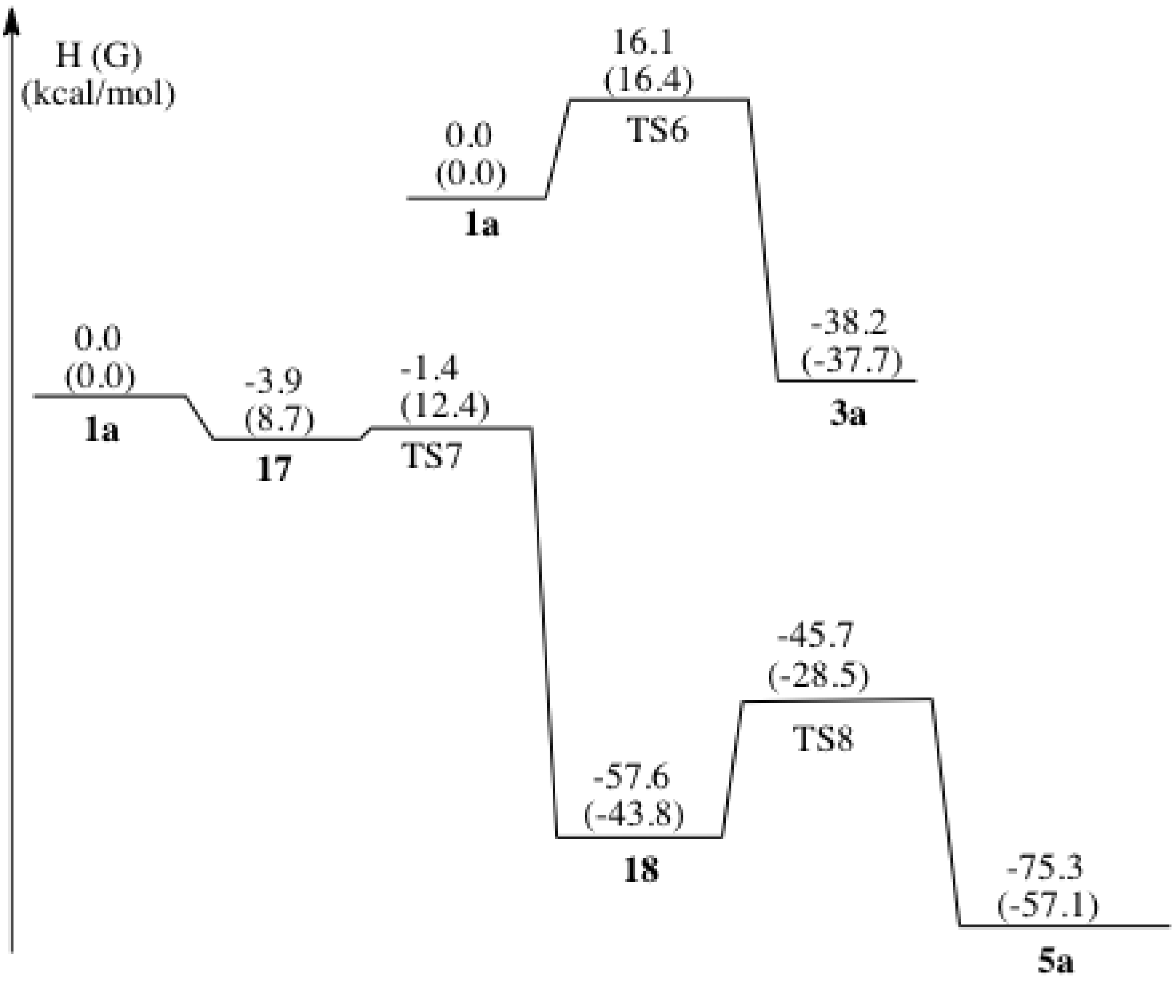

Figure SI3. Free activation energies for the radical transformations of thiobenzophenone *S*-methanides to thiirane **3a**, diradical **18**, and 1,4-dithiane **5a**, respectively. Computed enthalpies (free energies in parentheses) at 298K at the SMD(tetrahydrofuran)/M06-2X/6-311+G(2df,p)//M06-2X/6-31G(d) level of theory.

Table SI1. Zero-point energies (kcal/mol), integrated heat capacities (kcal/mol, 298 K), entropies (kcal/mol·K), total energies (hartrees) with m06-2x and with the smd solvation model, relative enthalpies (kcal/mol, 298 K) in THF, solvation free energies (kcal/mol) in THF, and relative free energies (kcal/mol, 298 K) in THF

		ZPE/a	Cp/a	S/a	M06-2X/b	SMD/b	Δ*G*(solv)	Δ*H*	Δ*G*	$\langle S^2 \rangle^b$
**8**	se2a	114.35	8.65	116.35	-3262.893335	-3262.904925	-7.27	0.0	0.0	
**TS1**	se2d	112.77	8.52	114.35	-3262.866786	-3262.878115	-7.11	15.1	15.7	0.66
**3a**	se2c	116.25	8.13	112.32	-3262.950730	-3262.965540	-9.29	-36.7	-35.5	
2x**8**	two	228.70	17.30	232.70	-6525.786670	-6525.809850	-14.54	0.0	0.0	
**15**	se5	230.26	17.40	178.05	-6525.798432	-6525.819618	-13.29	-4.5	11.8	
**TS2**	se555	229.15	17.15	181.96	-6525.791919	-6525.815530	-14.82	-3.3	11.9	
**12E**	se99a	231.80	16.96	185.45	-6525.892830	-6525.917248	-15.32	-64.6	-50.5	1.04
**12H**	se222zz2	232.00	16.83	180.39	-6525.889133	-6525.915066	-16.27	-63.2	-47.6	1.03
**12I**	se88a	231.88	17.06	186.53	-6525.887779	-6525.911886	-15.13	-61.1	-47.3	1.05
**12F**	se222y	232.19	16.70	176.33	-6525.887327	-6525.914046	-16.77	-62.5	-45.7	1.02
**12G**	se888zz	232.46	16.74	176.90	-6525.879437	-6525.903646	-15.19	-55.7	-39.0	1.04
**TS3**	se222z1	232.20	16.16	169.46	-6525.876664	-6525.903960	-17.13	-56.7	-37.8	0.56
**9**	se2	235.17	16.01	168.77	-6525.930619	-6525.959888	-18.37	-89.0	-69.9	
**TS4**	se888z	232.52	16.16	169.67	-6525.864547	-6525.889072	-15.39	-47.0	-28.2	0.62
**13**	se81	235.23	15.79	165.56	-6525.922020	-6525.945460	-14.71	-80.1	-60.1	
**TS5**	se222zzz	232.58	16.15	168.06	-6525.862192	-6525.887558	-15.92	-46.0	-26.8	0.45
**14**	se222zz1	235.10	16.02	167.11	-6525.896821	-6525.923684	-16.86	-66.3	-46.8	
**1a**	se2b	136.26	8.59	113.03	-938.763768	-938.777876	-8.85	0.0	0.0	
**TS6**	seq2d	134.74	8.47	112.05	-938.735520	-938.749635	-8.86	16.1	16.4	0.59
**3a**	seq2c	138.02	8.16	111.20	-938.823274	-938.840879	-11.05	-38.2	-37.7	
2x**1a**	two	272.52	17.18	226.06	-1877.527536	-1877.555752	-17.70	0.0	0.0	
**17**	seq5	273.34	17.72	183.93	-1877.538980	-1877.564068	-15.74	-3.9	8.7	
**TS7**	seq555	273.19	17.11	179.72	-1877.532362	-1877.558888	-16.64	-1.4	12.4	
**18**	seq222y	275.92	16.92	180.02	-1877.622128	-1877.652463	-19.04	-57.6	-43.8	
**TS8**	seq888z	276.37	16.20	168.43	-1877.604152	-1877.633147	-18.19	-45.7	-28.5	0.60
**5a**	seq81	278.52	15.87	164.98	-1877.655275	-1877.683231	-17.54	-75.3	-57.1	

a) Basis set “a” is 6-31G(d), basis set “b” is 6-311+G(2df,p), SMD is the solvation model with parameters for tetrahydrofuran. Geometries were optimized at the M06-2X/6-31G(d) level, single-point energies were calculated with the 6-311+G(2df,p) basis set.

b) Spin-squared value at the M06-2X/6-31G(d) level.

Table SI2. Cartesian coordinates of relevant species at the M06-2X/6-31G(d) level

8

Standard orientation:

Center Number	Atomic Number	Atomic Type	Coordinates (Angstroms) X	Y	Z
1	6	0	-3.003766	-1.367688	0.403468
2	6	0	-2.453581	-1.263545	1.642635
3	6	0	-1.231223	-0.522718	1.691403
4	6	0	-0.792435	-0.030438	0.496567
5	1	0	-2.905873	-1.705049	2.523715
6	1	0	-0.673664	-0.353918	2.606546
7	6	0	0.415519	0.738396	0.220753
8	1	0	-3.919468	-1.877097	0.137630
9	6	0	1.719323	0.078677	0.105819
10	6	0	1.758398	-1.286727	-0.225621
11	6	0	2.936233	0.748608	0.312717
12	6	0	2.971499	-1.950285	-0.353267
13	1	0	0.822041	-1.809743	-0.396831
14	6	0	4.146243	0.082404	0.165576
15	1	0	2.932387	1.793144	0.612665
16	6	0	4.173444	-1.271186	-0.164312
17	1	0	2.976808	-3.005289	-0.610670
18	1	0	5.074790	0.620548	0.331267
19	1	0	5.120260	-1.791977	-0.264415
20	6	0	-1.227620	2.921236	0.061652
21	1	0	-2.058659	2.272234	0.304305
22	1	0	-1.366259	3.972172	-0.144478
23	34	0	-1.977997	-0.505735	-0.874555
24	16	0	0.298584	2.363724	-0.094326

TS1

Standard orientation:

Center Number	Atomic Number	Atomic Type	Coordinates (Angstroms) X	Y	Z
1	6	0	3.242190	-0.963676	-0.022073
2	6	0	3.363918	0.163849	-0.779110
3	6	0	2.155733	0.886485	-0.970174
4	6	0	1.034964	0.348436	-0.374798
5	1	0	4.306196	0.476297	-1.215451
6	1	0	2.095309	1.801416	-1.548731

7	6	0	-0.294010	0.901098	-0.348860
8	1	0	4.023888	-1.668326	0.225045
9	6	0	-1.498627	0.084415	-0.252058
10	6	0	-1.504387	-1.253136	-0.702703
11	6	0	-2.701162	0.610863	0.258994
12	6	0	-2.654996	-2.024977	-0.624153
13	1	0	-0.606780	-1.671758	-1.143959
14	6	0	-3.845935	-0.169815	0.338801
15	1	0	-2.726063	1.640029	0.602151
16	6	0	-3.830848	-1.492823	-0.098290
17	1	0	-2.636585	-3.046563	-0.991100
18	1	0	-4.756432	0.257988	0.747014
19	1	0	-4.727836	-2.100718	-0.036716
20	6	0	-0.021599	2.617064	1.329427
21	1	0	0.945947	2.960210	1.676294
22	1	0	-0.648848	2.034454	1.993651
23	34	0	1.503238	-1.222605	0.553571
24	16	0	-0.440646	2.664928	-0.353728

3a

Standard orientation:

Center Number	Atomic Number	Atomic Type	Coordinates (Angstroms) X	Y	Z
1	6	0	-3.155655	-0.952439	0.492277
2	6	0	-2.770514	-0.353800	1.650416
3	6	0	-1.541934	0.375017	1.565491
4	6	0	-0.952288	0.371776	0.340401
5	1	0	-3.350579	-0.419462	2.564143
6	1	0	-1.103828	0.907434	2.403242
7	6	0	0.368334	0.930930	-0.061613
8	1	0	-4.047150	-1.539365	0.322633
9	6	0	1.546917	-0.011434	0.046787
10	6	0	1.346994	-1.366111	0.323897
11	6	0	2.852136	0.447602	-0.163199
12	6	0	2.429969	-2.240757	0.387591
13	1	0	0.342037	-1.744640	0.474549
14	6	0	3.928744	-0.427963	-0.104453
15	1	0	3.025053	1.504242	-0.347180
16	6	0	3.722833	-1.778293	0.173598
17	1	0	2.254166	-3.289323	0.607293
18	1	0	4.934240	-0.051931	-0.266708
19	1	0	4.565233	-2.460873	0.225746
20	6	0	0.356312	1.903043	-1.194444
21	1	0	-0.605013	2.115483	-1.655925
22	1	0	1.207537	1.886177	-1.870533
23	34	0	-1.947963	-0.625146	-0.875261
24	16	0	0.638623	2.685114	0.410069

15

Standard orientation:

Center Number	Atomic Number	Atomic Type	Coordinates (Angstroms) X	Y	Z

1	6	0	1.588431	-0.371031	3.351796
2	6	0	2.345762	0.683234	2.943579
3	6	0	2.566532	0.745300	1.538190
4	6	0	1.994228	-0.259391	0.795532
5	1	0	2.765050	1.408085	3.632802
6	1	0	3.155595	1.523644	1.064690
7	6	0	-2.566532	-0.745300	1.538190
8	6	0	-2.345762	-0.683234	2.943579
9	6	0	-1.588431	0.371031	3.351796
10	1	0	-3.155595	-1.523644	1.064690
11	1	0	-2.765050	-1.408085	3.632802
12	1	0	-1.298755	0.625634	4.361529
13	6	0	-1.994228	0.259391	0.795532
14	6	0	-2.049404	0.364170	-0.649226
15	6	0	2.049404	-0.364170	-0.649226
16	1	0	1.298755	-0.625634	4.361529
17	6	0	-2.049404	-0.856160	-1.479874
18	6	0	-2.842798	-0.984521	-2.629336
19	6	0	-1.217437	-1.930553	-1.125293
20	6	0	-2.778797	-2.131928	-3.414125
21	1	0	-3.531035	-0.188103	-2.897056
22	6	0	-1.168571	-3.080319	-1.900656
23	1	0	-0.594826	-1.833383	-0.241293
24	6	0	-1.946001	-3.186846	-3.053791
25	1	0	-3.401950	-2.207923	-4.300083
26	1	0	-0.503310	-3.887098	-1.606640
27	1	0	-1.907355	-4.086209	-3.660193
28	6	0	2.049404	0.856160	-1.479874
29	6	0	2.842798	0.984521	-2.629336
30	6	0	1.217437	1.930553	-1.125293
31	6	0	2.778797	2.131928	-3.414125
32	1	0	3.531035	0.188103	-2.897056
33	6	0	1.168571	3.080319	-1.900656
34	1	0	0.594826	1.833383	-0.241293
35	6	0	1.946001	3.186846	-3.053791
36	1	0	3.401950	2.207923	-4.300083
37	1	0	0.503310	3.887098	-1.606640
38	1	0	1.907355	4.086209	-3.660193
39	6	0	-2.361299	3.128150	-0.575323
40	1	0	-2.398367	4.053771	-1.131883
41	1	0	-2.452923	3.117840	0.500885
42	6	0	2.361299	-3.128150	-0.575323
43	1	0	2.452923	-3.117840	0.500885
44	1	0	2.398367	-4.053771	-1.131883
45	34	0	1.022571	-1.401394	1.923182
46	34	0	-1.022571	1.401394	1.923182
47	16	0	-2.192628	1.787023	-1.494988
48	16	0	2.192628	-1.787023	-1.494988

TS2

Standard orientation:

Center Number	Atomic Number	Atomic Type	Coordinates (Angstroms) X	Y	Z
1	6	0	-0.787710	-0.182251	2.456071
2	6	0	0.246999	0.671364	2.708856

3	6	0	1.414279	0.455249	1.941081
4	6	0	1.356829	-0.597025	1.039445
5	1	0	0.178127	1.467637	3.442410
6	1	0	2.311966	1.049224	2.061199
7	6	0	-1.766526	2.159378	0.490048
8	6	0	-0.818427	3.196272	0.688474
9	6	0	0.274774	3.142378	-0.121146
10	1	0	-2.666117	2.066745	1.086701
11	1	0	-0.949716	3.965824	1.441829
12	1	0	1.111866	3.825846	-0.144603
13	6	0	-1.467640	1.252624	-0.510961
14	6	0	-2.292945	0.154234	-0.913147
15	6	0	2.412876	-0.992749	0.160271
16	1	0	-1.767611	-0.187715	2.912469
17	6	0	-3.640498	0.003346	-0.311920
18	6	0	-4.586291	1.036503	-0.399531
19	6	0	-3.995403	-1.174635	0.358229
20	6	0	-5.849181	0.888220	0.158090
21	1	0	-4.317036	1.951470	-0.919406
22	6	0	-5.266884	-1.325919	0.906933
23	1	0	-3.257691	-1.966705	0.460748
24	6	0	-6.196144	-0.295942	0.810317
25	1	0	-6.572228	1.693812	0.073629
26	1	0	-5.525175	-2.247828	1.418982
27	1	0	-7.185577	-0.410735	1.241788
28	6	0	3.707814	-0.254481	0.191500
29	6	0	4.906658	-0.902856	0.507170
30	6	0	3.747134	1.109407	-0.129580
31	6	0	6.114401	-0.208584	0.498641
32	1	0	4.885620	-1.954904	0.776434
33	6	0	4.949303	1.804307	-0.122839
34	1	0	2.819056	1.605552	-0.393876
35	6	0	6.139341	1.146608	0.188396
36	1	0	7.034304	-0.728870	0.747005
37	1	0	4.961467	2.860103	-0.376283
38	1	0	7.079048	1.689969	0.188577
39	6	0	-0.333697	-1.335634	-2.286546
40	1	0	-0.145424	-1.946579	-3.159229
41	1	0	0.461887	-0.713041	-1.903850
42	6	0	1.151951	-3.253799	-0.978362
43	1	0	0.369293	-3.327934	-0.242700
44	1	0	1.248541	-4.037957	-1.718545
45	34	0	-0.350646	-1.386622	1.128955
46	34	0	0.184896	1.702118	-1.283908
47	16	0	-1.941291	-1.079393	-1.992377
48	16	0	2.505967	-2.317912	-0.863988

12E

Standard orientation:

Center Number	Atomic Number	Atomic Type	Coordinates (Angstroms) X	Y	Z
1	6	0	5.152349	-0.284657	-1.005204
2	6	0	5.021502	0.958455	-0.447234
3	6	0	3.937097	1.101047	0.447207
4	6	0	3.154423	-0.041166	0.623692

5	1	0	5.706597	1.770625	-0.665506
6	1	0	3.733356	2.015390	0.991934
7	6	0	-4.076450	1.022757	-0.746526
8	6	0	-5.240712	0.878967	0.044402
9	6	0	-5.339376	-0.308493	0.716169
10	1	0	-3.888743	1.889957	-1.368623
11	1	0	-6.003984	1.647516	0.101672
12	1	0	-6.151802	-0.618727	1.358323
13	6	0	-3.199539	-0.058043	-0.719647
14	6	0	-1.947657	-0.225515	-1.361417
15	6	0	1.997838	-0.217590	1.409905
16	1	0	5.919481	-0.601465	-1.697872
17	6	0	-1.135850	0.885999	-1.843347
18	6	0	-1.235391	2.169185	-1.266630
19	6	0	-0.192909	0.706503	-2.877249
20	6	0	-0.469189	3.226490	-1.739909
21	1	0	-1.889211	2.320672	-0.414421
22	6	0	0.584631	1.762253	-3.330153
23	1	0	-0.094361	-0.272676	-3.335120
24	6	0	0.444617	3.033602	-2.773970
25	1	0	-0.574100	4.206580	-1.283234
26	1	0	1.297909	1.594382	-4.131132
27	1	0	1.046467	3.860674	-3.136955
28	6	0	1.206903	0.899242	1.927044
29	6	0	1.070278	2.090568	1.190519
30	6	0	0.524575	0.798062	3.154986
31	6	0	0.320445	3.150059	1.687053
32	1	0	1.523059	2.163841	0.205970
33	6	0	-0.225111	1.858745	3.642934
34	1	0	0.607092	-0.120159	3.727692
35	6	0	-0.325291	3.045082	2.916530
36	1	0	0.226590	4.057536	1.097732
37	1	0	-0.731543	1.761518	4.598316
38	1	0	-0.911228	3.874186	3.300586
39	6	0	0.162644	-1.883171	-0.666693
40	1	0	0.702962	-2.785419	-0.967913
41	1	0	0.764642	-1.017712	-0.965258
42	6	0	-0.100415	-1.885671	0.831898
43	1	0	-0.707872	-1.024114	1.132316
44	1	0	-0.628474	-2.794348	1.135019
45	34	0	3.849681	-1.458847	-0.406751
46	34	0	-3.899248	-1.421614	0.377635
47	16	0	-1.401617	-1.886737	-1.614162
48	16	0	1.476740	-1.868817	1.765428

12H

Standard orientation:

Center Number	Atomic Number	Atomic Type	Coordinates (Angstroms) X	Y	Z
1	6	0	-2.077339	-0.098315	2.634111
2	6	0	-1.131429	-1.031119	2.957230
3	6	0	-1.198727	-1.490606	0.628593
4	1	0	-0.776527	-1.174541	3.972330
5	1	0	0.180087	-2.495744	1.966364
6	6	0	1.131429	1.031119	2.957230

7	6	0	2.077339	0.098315	2.634111
8	1	0	-0.180087	2.495744	1.966364
9	1	0	0.776527	1.174541	3.972330
10	1	0	2.574498	-0.583578	3.309885
11	6	0	1.010318	2.164485	-0.596369
12	6	0	-1.010318	-2.164485	-0.596369
13	1	0	-2.574498	0.583578	3.309885
14	6	0	0.382227	3.487107	-0.669243
15	6	0	0.583934	4.443791	0.344543
16	6	0	-0.412874	3.853398	-1.770604
17	6	0	-0.009405	5.696975	0.271448
18	1	0	1.239845	4.202749	1.174681
19	6	0	-1.010318	5.104809	-1.835698
20	1	0	-0.561650	3.138718	-2.573606
21	6	0	-0.816119	6.032807	-0.814202
22	1	0	0.171495	6.421354	1.059775
23	1	0	-1.631029	5.357814	-2.689749
24	1	0	-1.279354	7.012829	-0.869537
25	6	0	-0.382227	-3.487107	-0.669243
26	6	0	-0.583934	-4.443791	0.344543
27	6	0	0.412874	-3.853398	-1.770604
28	6	0	0.009405	-5.696975	0.271448
29	1	0	-1.239845	-4.202749	1.174681
30	6	0	1.010318	-5.104809	-1.835698
31	1	0	0.561650	-3.138718	-2.573606
32	6	0	0.816119	-6.032807	-0.814202
33	1	0	-0.171495	-6.421354	1.059775
34	1	0	1.631029	-5.357814	-2.689749
35	1	0	1.279354	-7.012829	-0.869537
36	6	0	0.750646	-0.107358	-2.197847
37	1	0	1.089246	-0.574132	-3.127445
38	1	0	1.039056	-0.753255	-1.363265
39	6	0	-0.750646	0.107358	-2.197847
40	1	0	-1.039056	0.753255	-1.363265
41	1	0	-1.089246	0.574132	-3.127445
42	34	0	-2.435886	-0.081686	0.816987
43	34	0	2.435886	0.081686	0.816987
44	16	0	1.646714	1.486849	-2.097112
45	16	0	-1.646714	-1.486849	-2.097112
46	6	0	-0.615958	-1.766838	1.867506
47	6	0	0.615958	1.766838	1.867506
48	6	0	1.198727	1.490606	0.628593

12I

Standard orientation:

Center Number	Atomic Number	Atomic Type	Coordinates (Angstroms) X	Y	Z
1	6	0	2.063949	-2.208462	0.068117
2	6	0	-2.063949	2.208462	0.068117
3	6	0	3.426476	-2.553290	0.193548
4	6	0	-3.426476	2.553290	0.193548
5	6	0	-0.451785	0.613276	-1.570193
6	16	0	-1.675821	0.516010	-0.210717
7	6	0	0.451785	-0.613276	-1.570193
8	16	0	1.675821	-0.516010	-0.210717

9	1	0	-0.994302	0.668587	-2.517270
10	1	0	0.129604	1.532294	-1.452732
11	1	0	-0.129604	-1.532294	-1.452732
12	1	0	0.994302	-0.668587	-2.517270
13	34	0	-4.799323	1.418371	-0.425981
14	34	0	4.799323	-1.418371	-0.425981
15	6	0	-6.013953	2.684528	0.168708
16	6	0	-3.983219	3.696787	0.767223
17	6	0	3.983219	-3.696787	0.767223
18	6	0	6.013953	-2.684528	0.168708
19	6	0	5.394925	-3.761868	0.740927
20	6	0	-5.394925	3.761868	0.740927
21	1	0	-7.079025	2.533378	0.062902
22	1	0	-5.946589	4.597993	1.157007
23	1	0	-3.363651	4.456141	1.229685
24	1	0	7.079025	-2.533378	0.062902
25	1	0	5.946589	-4.597993	1.157007
26	1	0	3.363651	-4.456141	1.229685
27	6	0	0.984826	-3.178445	0.261688
28	6	0	1.111668	-4.499637	-0.206427
29	6	0	-0.226837	-2.804601	0.872327
30	6	0	0.077358	-5.413199	-0.046711
31	1	0	2.021687	-4.793180	-0.720524
32	6	0	-1.258497	-3.721521	1.026005
33	1	0	-0.344164	-1.787151	1.235328
34	6	0	-1.111668	-5.031364	0.572020
35	1	0	0.195240	-6.425596	-0.421079
36	1	0	-2.181018	-3.410493	1.506583
37	1	0	-1.919894	-5.745885	0.691518
38	6	0	-0.984826	3.178445	0.261688
39	6	0	-1.111668	4.499637	-0.206427
40	6	0	0.226837	2.804601	0.872327
41	6	0	-0.077358	5.413199	-0.046711
42	1	0	-2.021687	4.793180	-0.720524
43	6	0	1.258497	3.721521	1.026005
44	1	0	0.344164	1.787151	1.235328
45	6	0	1.111668	5.031364	0.572020
46	1	0	-0.195240	6.425596	-0.421079
47	1	0	2.181018	3.410493	1.506583
48	1	0	1.919894	5.745885	0.691518

12F

Standard orientation:

Center Number	Atomic Number	Atomic Type	Coordinates (Angstroms) X	Y	Z
1	6	0	-0.946102	-1.136513	3.096681
2	6	0	-0.030872	-2.155197	3.025593
3	6	0	0.172277	-2.685468	1.735385
4	6	0	-0.629122	-2.134294	0.726836
5	1	0	0.499618	-2.524155	3.897225
6	1	0	0.879694	-3.478927	1.524880
7	6	0	-0.172277	2.685468	1.735385
8	6	0	0.030872	2.155197	3.025593
9	6	0	0.946102	1.136513	3.096681
10	1	0	-0.879694	3.478927	1.524880

11	1	0	-0.499618	2.524155	3.897225
12	1	0	1.242897	0.588152	3.978954
13	6	0	0.629122	2.134294	0.726836
14	6	0	0.738532	2.537454	-0.616644
15	6	0	-0.738532	-2.537454	-0.616644
16	1	0	-1.242897	-0.588152	3.978954
17	6	0	0.256624	3.844191	-1.078739
18	6	0	0.363875	4.984662	-0.260108
19	6	0	-0.296386	4.004206	-2.361965
20	6	0	-0.090291	6.222117	-0.697211
21	1	0	0.838382	4.893934	0.711700
22	6	0	-0.755067	5.241898	-2.792343
23	1	0	-0.368291	3.141102	-3.015709
24	6	0	-0.658747	6.357227	-1.962240
25	1	0	0.014278	7.089027	-0.051932
26	1	0	-1.190763	5.336659	-3.782237
27	1	0	-1.013094	7.325051	-2.302629
28	6	0	-0.256624	-3.844191	-1.078739
29	6	0	-0.363875	-4.984662	-0.260108
30	6	0	0.296386	-4.004206	-2.361965
31	6	0	0.090291	-6.222117	-0.697211
32	1	0	-0.838382	-4.893934	0.711700
33	6	0	0.755067	-5.241898	-2.792343
34	1	0	0.368291	-3.141102	-3.015709
35	6	0	0.658747	-6.357227	-1.962240
36	1	0	-0.014278	-7.089027	-0.051932
37	1	0	1.190763	-5.336659	-3.782237
38	1	0	1.013094	-7.325051	-2.302629
39	6	0	0.755067	-0.071510	-1.729487
40	1	0	1.096532	-0.590531	-2.630062
41	1	0	1.085038	-0.646256	-0.861783
42	6	0	-0.755067	0.071510	-1.729487
43	1	0	-1.085038	0.646256	-0.861783
44	1	0	-1.096532	0.590531	-2.630062
45	34	0	-1.634222	-0.708713	1.436215
46	34	0	1.634222	0.708713	1.436215
47	16	0	1.602105	1.554740	-1.800893
48	16	0	-1.602105	-1.554740	-1.800893

12G

Standard orientation:

Center Number	Atomic Number	Atomic Type	Coordinates (Angstroms) X	Y	Z
1	6	0	1.532077	-0.839405	-0.383866
2	6	0	-1.532077	0.839405	-0.383866
3	6	0	1.399140	-2.242731	-0.720057
4	6	0	-1.399140	2.242731	-0.720057
5	6	0	-0.690134	0.325498	-3.023926
6	16	0	-1.821214	-0.271541	-1.723586
7	6	0	0.690134	-0.325498	-3.023926
8	16	0	1.821214	0.271541	-1.723586
9	1	0	-1.194523	0.077147	-3.963642
10	1	0	-0.618366	1.413932	-2.957040
11	1	0	0.618366	-1.413932	-2.957040
12	1	0	1.194523	-0.077147	-3.963642

13	34	0	-0.004913	3.338667	-0.111033
14	34	0	0.004913	-3.338667	-0.111033
15	6	0	-0.718611	4.709764	-1.130610
16	6	0	-2.187981	2.946316	-1.599800
17	6	0	2.187981	-2.946316	-1.599800
18	6	0	0.718611	-4.709764	-1.130610
19	6	0	1.821214	-4.304738	-1.819016
20	6	0	-1.821214	4.304738	-1.819016
21	1	0	-0.271603	5.694062	-1.137882
22	1	0	-2.383114	4.964139	-2.471262
23	1	0	-3.044270	2.476839	-2.073711
24	1	0	0.271603	-5.694062	-1.137882
25	1	0	2.383114	-4.964139	-2.471262
26	1	0	3.044270	-2.476839	-2.073711
27	6	0	1.640815	-0.325320	0.962159
28	6	0	1.477509	-1.155847	2.095944
29	6	0	1.967957	1.029868	1.198492
30	6	0	1.586794	-0.640544	3.377933
31	1	0	1.253178	-2.208508	1.969656
32	6	0	2.071405	1.536187	2.484885
33	1	0	2.149119	1.689061	0.358749
34	6	0	1.881424	0.706679	3.588179
35	1	0	1.438219	-1.302468	4.225989
36	1	0	2.315945	2.585720	2.623618
37	1	0	1.961457	1.101423	4.596104
38	6	0	-1.640815	0.325320	0.962159
39	6	0	-1.477509	1.155847	2.095944
40	6	0	-1.967957	-1.029868	1.198492
41	6	0	-1.586794	0.640544	3.377933
42	1	0	-1.253178	2.208508	1.969656
43	6	0	-2.071405	-1.536187	2.484885
44	1	0	-2.149119	-1.689061	0.358749
45	6	0	-1.881424	-0.706679	3.588179
46	1	0	-1.438219	1.302468	4.225989
47	1	0	-2.315945	-2.585720	2.623618
48	1	0	-1.961457	-1.101423	4.596104

TS3

Standard orientation:

Center Number	Atomic Number	Atomic Type	Coordinates (Angstroms) X	Y	Z
1	6	0	-0.896186	-0.853365	3.036432
2	6	0	-0.068639	-1.982477	3.009403
3	6	0	0.123534	-2.554977	1.767472
4	6	0	-0.676289	-2.017180	0.711699
5	1	0	0.436641	-2.338908	3.901689
6	1	0	0.808544	-3.374507	1.585530
7	6	0	-0.123534	2.554977	1.767472
8	6	0	0.068639	1.982477	3.009403
9	6	0	0.896186	0.853365	3.036432
10	1	0	-0.808544	3.374507	1.585530
11	1	0	-0.436641	2.338908	3.901689
12	1	0	1.302445	0.387521	3.922251
13	6	0	0.676289	2.017180	0.711699
14	6	0	0.784654	2.452553	-0.589614

15	6	0	-0.784654	-2.452553	-0.589614
16	1	0	-1.302445	-0.387521	3.922251
17	6	0	0.253679	3.755976	-1.030238
18	6	0	0.381709	4.892127	-0.213534
19	6	0	-0.363304	3.906441	-2.281909
20	6	0	-0.120300	6.122776	-0.619909
21	1	0	0.906531	4.804890	0.732964
22	6	0	-0.868496	5.136259	-2.683796
23	1	0	-0.443540	3.044923	-2.937161
24	6	0	-0.753815	6.249792	-1.853801
25	1	0	-0.001610	6.989263	0.023483
26	1	0	-1.353038	5.226558	-3.651151
27	1	0	-1.143652	7.211480	-2.172178
28	6	0	-0.253679	-3.755976	-1.030238
29	6	0	-0.381709	-4.892127	-0.213534
30	6	0	0.363304	-3.906441	-2.281909
31	6	0	0.120300	-6.122776	-0.619909
32	1	0	-0.906531	-4.804890	0.732964
33	6	0	0.868496	-5.136259	-2.683796
34	1	0	0.443540	-3.044923	-2.937161
35	6	0	0.753815	-6.249792	-1.853801
36	1	0	0.001610	-6.989263	0.023483
37	1	0	1.353038	-5.226558	-3.651151
38	1	0	1.143652	-7.211480	-2.172178
39	6	0	0.753815	-0.097179	-1.813368
40	1	0	1.082732	-0.589688	-2.733137
41	1	0	1.058813	-0.715616	-0.967670
42	6	0	-0.753815	0.097179	-1.813368
43	1	0	-1.058813	0.715616	-0.967670
44	1	0	-1.082732	0.589688	-2.733137
45	34	0	-1.633007	-0.518927	1.367407
46	34	0	1.633007	0.518927	1.367407
47	16	0	1.664149	1.499230	-1.801844
48	16	0	-1.664149	-1.499230	-1.801844

9

Standard orientation:

Center Number	Atomic Number	Atomic Type	Coordinates (Angstroms) X	Y	Z
1	6	0	-0.499452	-0.594665	2.963894
2	6	0	0.217591	-1.913060	2.907804
3	6	0	0.163942	-2.607655	1.766067
4	6	0	-0.679620	-2.065143	0.698846
5	1	0	0.833582	-2.214009	3.751452
6	1	0	0.737749	-3.512323	1.596542
7	6	0	-0.163942	2.607655	1.766067
8	6	0	-0.217591	1.913060	2.907804
9	6	0	0.499452	0.594665	2.963894
10	1	0	-0.737749	3.512323	1.596542
11	1	0	-0.833582	2.214009	3.751452
12	1	0	1.156015	0.512556	3.836973
13	6	0	0.679620	2.065143	0.698846
14	6	0	0.757006	2.509903	-0.575776
15	6	0	-0.757006	-2.509903	-0.575776
16	1	0	-1.156015	-0.512556	3.836973

17	6	0	0.198170	3.819010	-0.992912
18	6	0	0.446746	4.966842	-0.228324
19	6	0	-0.568419	3.944227	-2.158334
20	6	0	-0.085937	6.197607	-0.598792
21	1	0	1.081260	4.887522	0.650135
22	6	0	-1.102590	5.173085	-2.526399
23	1	0	-0.741754	3.066508	-2.773471
24	6	0	-0.866387	6.303933	-1.746668
25	1	0	0.122321	7.077061	0.002830
26	1	0	-1.703686	5.249618	-3.427204
27	1	0	-1.278463	7.264461	-2.039760
28	6	0	-0.198170	-3.819010	-0.992912
29	6	0	-0.446746	-4.966842	-0.228324
30	6	0	0.568419	-3.944227	-2.158334
31	6	0	0.085937	-6.197607	-0.598792
32	1	0	-1.081260	-4.887522	0.650135
33	6	0	1.102590	-5.173085	-2.526399
34	1	0	0.741754	-3.066508	-2.773471
35	6	0	0.866387	-6.303933	-1.746668
36	1	0	-0.122321	-7.077061	0.002830
37	1	0	1.703686	-5.249618	-3.427204
38	1	0	1.278463	-7.264461	-2.039760
39	6	0	0.757006	-0.053280	-1.840850
40	1	0	1.114099	-0.523097	-2.762248
41	1	0	1.093753	-0.649346	-0.991313
42	6	0	-0.757006	0.053280	-1.840850
43	1	0	-1.093753	0.649346	-0.991313
44	1	0	-1.114099	0.523097	-2.762248
45	34	0	-1.601431	-0.541750	1.344379
46	34	0	1.601431	0.541750	1.344379
47	16	0	1.595334	1.582816	-1.847759
48	16	0	-1.595334	-1.582816	-1.847759

TS4

Standard orientation:

Center Number	Atomic Number	Atomic Type	Coordinates (Angstroms) X	Y	Z
1	6	0	1.095572	0.825768	-0.455892
2	6	0	-1.095572	-0.825768	-0.455892
3	6	0	2.235558	-0.064535	-0.684230
4	6	0	-2.235558	0.064535	-0.684230
5	6	0	-0.576748	-0.495962	-3.197261
6	16	0	-0.573030	-1.753790	-1.876736
7	6	0	0.576748	0.495962	-3.197261
8	16	0	0.573030	1.753790	-1.876736
9	1	0	-0.511646	-1.095065	-4.111977
10	1	0	-1.537257	0.022881	-3.200918
11	1	0	1.537257	-0.022881	-3.200918
12	1	0	0.511646	1.095065	-4.111977
13	34	0	-2.466964	1.758253	0.076121
14	34	0	2.466964	-1.758253	0.076121
15	6	0	-4.083457	1.875473	-0.811606
16	6	0	-3.306265	-0.219850	-1.500783
17	6	0	3.306265	0.219850	-1.500783
18	6	0	4.083457	-1.875473	-0.811606

19	6	0	4.324639	-0.771012	-1.569776
20	6	0	-4.324639	0.771012	-1.569776
21	1	0	-4.714105	2.747628	-0.709958
22	1	0	-5.221735	0.647513	-2.166393
23	1	0	-3.363235	-1.164816	-2.031889
24	1	0	4.714105	-2.747628	-0.709958
25	1	0	5.221735	-0.647513	-2.166393
26	1	0	3.363235	1.164816	-2.031889
27	6	0	0.896963	1.461129	0.846417
28	6	0	1.294721	0.828885	2.044383
29	6	0	0.336459	2.752188	0.959539
30	6	0	1.095572	1.434090	3.277753
31	1	0	1.768352	-0.145686	2.017204
32	6	0	0.139640	3.351275	2.195316
33	1	0	0.046216	3.296552	0.067506
34	6	0	0.508007	2.693300	3.366984
35	1	0	1.400686	0.907375	4.176941
36	1	0	-0.297645	4.344194	2.240587
37	1	0	0.353167	3.162230	4.333495
38	6	0	-0.896963	-1.461129	0.846417
39	6	0	-1.294721	-0.828885	2.044383
40	6	0	-0.336459	-2.752188	0.959539
41	6	0	-1.095572	-1.434090	3.277753
42	1	0	-1.768352	0.145686	2.017204
43	6	0	-0.139640	-3.351275	2.195316
44	1	0	-0.046216	-3.296552	0.067506
45	6	0	-0.508007	-2.693300	3.366984
46	1	0	-1.400686	-0.907375	4.176941
47	1	0	0.297645	-4.344194	2.240587
48	1	0	-0.353167	-3.162230	4.333495

13

Standard orientation:

Center Number	Atomic Number	Atomic Type	Coordinates (Angstroms) X	Y	Z
1	6	0	0.430384	0.103660	-0.738437
2	6	0	-0.189873	-0.709435	0.508273
3	6	0	1.882574	0.548167	-0.601860
4	6	0	-1.614042	-1.180977	0.231692
5	6	0	0.691073	-3.188817	-0.644620
6	16	0	0.850699	-2.211449	0.880979
7	6	0	1.211308	-2.465445	-1.873946
8	16	0	0.271202	-0.970460	-2.271526
9	1	0	1.282793	-4.090651	-0.453715
10	1	0	-0.353967	-3.479336	-0.783064
11	1	0	2.274871	-2.232702	-1.791948
12	1	0	1.072335	-3.091758	-2.761846
13	34	0	-2.924825	-0.116155	-0.573837
14	34	0	3.328667	-0.544333	-0.091417
15	6	0	-4.167407	-1.422532	-0.146218
16	6	0	-2.188700	-2.318377	0.724725
17	6	0	2.355740	1.789149	-0.931303
18	6	0	4.456634	0.893958	-0.413791
19	6	0	3.767050	1.985991	-0.825562
20	6	0	-3.593157	-2.465819	0.505210

21	1	0	-5.205075	-1.322391	-0.434002
22	1	0	-4.145494	-3.338329	0.836369
23	1	0	-1.610161	-3.052529	1.273829
24	1	0	5.522076	0.814174	-0.246280
25	1	0	4.236663	2.937210	-1.050771
26	1	0	1.699028	2.581385	-1.267406
27	6	0	-0.404220	1.375158	-1.035917
28	6	0	-0.608539	2.326275	-0.023810
29	6	0	-0.940943	1.653402	-2.298796
30	6	0	-1.394576	3.451814	-0.241934
31	1	0	-0.129536	2.210507	0.938762
32	6	0	-1.720128	2.786185	-2.519984
33	1	0	-0.781954	0.980742	-3.131902
34	6	0	-1.974681	3.678710	-1.486489
35	1	0	-1.546534	4.155626	0.570393
36	1	0	-2.133927	2.959051	-3.508492
37	1	0	-2.594813	4.553835	-1.654079
38	6	0	-0.151088	0.053034	1.859430
39	6	0	-1.338904	0.385820	2.525083
40	6	0	1.061439	0.417922	2.464529
41	6	0	-1.316278	1.067210	3.737977
42	1	0	-2.297686	0.147745	2.082931
43	6	0	1.077665	1.101031	3.677525
44	1	0	2.009759	0.205841	1.988696
45	6	0	-0.108778	1.430527	4.323541
46	1	0	-2.255995	1.314426	4.222334
47	1	0	2.033114	1.372922	4.115509
48	1	0	-0.091906	1.961709	5.270166

TS5

Standard orientation:

Center Number	Atomic Number	Atomic Type	Coordinates (Angstroms) X	Y	Z
1	6	0	1.872141	2.269514	-1.685906
2	6	0	1.389056	1.267809	-2.452974
3	6	0	1.609935	-0.002766	-0.415177
4	1	0	1.148620	1.400727	-3.503600
5	1	0	1.056300	-0.899852	-2.335485
6	6	0	-1.389056	-1.267809	-2.452974
7	6	0	-1.872141	-2.269514	-1.685906
8	1	0	-1.056300	0.899852	-2.335485
9	1	0	-1.148620	-1.400727	-3.503600
10	1	0	-2.064847	-3.287445	-1.996511
11	6	0	-1.609935	1.055430	0.471497
12	6	0	1.609935	-1.055430	0.471497
13	1	0	2.064847	3.287445	-1.996511
14	6	0	-1.339574	2.443800	0.050253
15	6	0	-1.835136	2.935415	-1.170452
16	6	0	-0.594681	3.320252	0.852159
17	6	0	-1.505629	4.206619	-1.618791
18	1	0	-2.505810	2.314568	-1.755970
19	6	0	-0.257964	4.591443	0.402698
20	1	0	-0.270674	2.995742	1.834831
21	6	0	-0.689814	5.032513	-0.845215
22	1	0	-1.900404	4.562266	-2.565683

23	1	0	0.344491	5.239356	1.032190
24	1	0	-0.427366	6.025878	-1.196110
25	6	0	1.339574	-2.443800	0.050253
26	6	0	1.835136	-2.935415	-1.170452
27	6	0	0.594681	-3.320252	0.852159
28	6	0	1.505629	-4.206619	-1.618791
29	1	0	2.505810	-2.314568	-1.755970
30	6	0	0.257964	-4.591443	0.402698
31	1	0	0.270674	-2.995742	1.834831
32	6	0	0.689814	-5.032513	-0.845215
33	1	0	1.900404	-4.562266	-2.565683
34	1	0	-0.344491	-5.239356	1.032190
35	1	0	0.427366	-6.025878	-1.196110
36	6	0	-0.693631	-0.324835	2.733287
37	1	0	-0.981930	-0.648139	3.737645
38	1	0	-0.707295	-1.205099	2.092856
39	6	0	0.693631	0.324835	2.733287
40	1	0	0.707295	1.205099	2.092856
41	1	0	0.981930	0.648139	3.737645
42	34	0	2.224438	1.728564	0.070968
43	34	0	-2.224438	-1.728564	0.070968
44	16	0	-2.034665	0.796225	2.186968
45	16	0	2.034665	-0.796225	2.186968
46	6	0	1.142730	0.019680	-1.779474
47	6	0	-1.142730	-0.019680	-1.779474
48	6	0	-1.609935	0.002766	-0.415177

14

Standard orientation:

Center Number	Atomic Number	Atomic Type	Coordinates (Angstroms) X	Y	Z
1	6	0	-0.134058	2.686133	2.101897
2	6	0	-0.550434	1.555650	2.670814
3	6	0	-1.019944	0.975821	0.367068
4	1	0	-0.665659	1.436205	3.743966
5	1	0	-1.466205	-0.328316	2.023527
6	6	0	0.550434	-1.555650	2.670814
7	6	0	0.134058	-2.686133	2.101897
8	1	0	1.466205	0.328316	2.023527
9	1	0	0.665659	-1.436205	3.743966
10	1	0	-0.120396	-3.611319	2.603020
11	6	0	1.879390	-0.496334	-0.551249
12	6	0	-1.879390	0.496334	-0.551249
13	1	0	0.120396	3.611319	2.603020
14	6	0	2.913173	0.523238	-0.229484
15	6	0	3.703238	0.358158	0.915343
16	6	0	3.165246	1.620627	-1.059950
17	6	0	4.678996	1.291411	1.250129
18	1	0	3.554619	-0.523901	1.532959
19	6	0	4.140286	2.553520	-0.727975
20	1	0	2.596582	1.737843	-1.977310
21	6	0	4.895301	2.397274	0.432556
22	1	0	5.282356	1.144080	2.140588
23	1	0	4.313878	3.403813	-1.380209
24	1	0	5.660125	3.124115	0.688045

25	6	0	-2.913173	-0.523238	-0.229484
26	6	0	-3.703238	-0.358158	0.915343
27	6	0	-3.165246	-1.620627	-1.059950
28	6	0	-4.678996	-1.291411	1.250129
29	1	0	-3.554619	0.523901	1.532959
30	6	0	-4.140286	-2.553520	-0.727975
31	1	0	-2.596582	-1.737843	-1.977310
32	6	0	-4.895301	-2.397274	0.432556
33	1	0	-5.282356	-1.144080	2.140588
34	1	0	-4.313878	-3.403813	-1.380209
35	1	0	-5.660125	-3.124115	0.688045
36	6	0	0.152201	-0.750401	-2.693155
37	1	0	0.009605	-1.184523	-3.686764
38	1	0	-0.514846	-1.278682	-2.010343
39	6	0	-0.152201	0.750401	-2.693155
40	1	0	0.514846	1.278682	-2.010343
41	1	0	-0.009605	1.184523	-3.686764
42	34	0	-0.019971	2.585703	0.196272
43	34	0	0.019971	-2.585703	0.196272
44	16	0	1.879390	-1.131527	-2.222146
45	16	0	-1.879390	1.131527	-2.222146
46	6	0	-0.699975	0.387585	1.724602
47	6	0	0.699975	-0.387585	1.724602
48	6	0	1.019944	-0.975821	0.367068

1a

Standard orientation:

Center Number	Atomic Number	Atomic Type	Coordinates (Angstroms) X	Y	Z
1	6	0	0.027570	0.667294	-0.025495
2	6	0	1.357435	0.035110	-0.044207
3	6	0	1.533486	-1.196483	-0.700996
4	6	0	2.478996	0.619242	0.567363
5	6	0	2.780538	-1.803813	-0.756092
6	1	0	0.679109	-1.666166	-1.179126
7	6	0	3.730673	0.018541	0.490226
8	1	0	2.362100	1.541280	1.129225
9	6	0	3.889924	-1.197209	-0.168145
10	1	0	2.889618	-2.753103	-1.272062
11	1	0	4.580806	0.493331	0.970856
12	1	0	4.864292	-1.672983	-0.214177
13	6	0	-1.418824	3.043704	0.168063
14	1	0	-2.335841	2.503239	0.354646
15	1	0	-1.406843	4.121608	0.099767
16	16	0	0.028922	2.324853	-0.069690
17	6	0	-1.197509	-0.147579	0.037430
18	6	0	-2.316827	0.129722	-0.761283
19	6	0	-1.251438	-1.259565	0.892552
20	6	0	-3.452362	-0.670030	-0.700322
21	1	0	-2.287032	0.972770	-1.446381
22	6	0	-2.387456	-2.057963	0.952361
23	1	0	-0.387952	-1.490403	1.509802
24	6	0	-3.495037	-1.765546	0.159200
25	1	0	-4.304159	-0.441143	-1.333648
26	1	0	-2.408922	-2.910683	1.624214

27	1	0	-4.382936	-2.387946	0.208002

TS6

Standard orientation:

Center Number	Atomic Number	Atomic Type	Coordinates (Angstroms) X	Y	Z
1	6	0	-0.033011	0.650950	-0.214351
2	6	0	-1.313883	-0.036354	-0.134543
3	6	0	-1.418929	-1.410310	-0.446929
4	6	0	-2.497248	0.647994	0.209632
5	6	0	-2.644991	-2.057314	-0.416354
6	1	0	-0.531056	-1.956101	-0.747934
7	6	0	-3.719642	-0.009397	0.247615
8	1	0	-2.444414	1.702622	0.459270
9	6	0	-3.803167	-1.364553	-0.063326
10	1	0	-2.699376	-3.109688	-0.678009
11	1	0	-4.613274	0.541061	0.525528
12	1	0	-4.760163	-1.875743	-0.035041
13	6	0	0.454424	2.499355	1.241542
14	1	0	1.445662	2.826218	1.533773
15	1	0	-0.195603	2.041841	1.977194
16	16	0	-0.008480	2.397113	-0.422840
17	6	0	1.248946	-0.062820	-0.091688
18	6	0	1.393088	-1.143614	0.796639
19	6	0	2.363470	0.314922	-0.858638
20	6	0	2.600000	-1.822338	0.905524
21	1	0	0.550244	-1.433457	1.417496
22	6	0	3.570485	-0.363703	-0.744930
23	1	0	2.268210	1.145227	-1.551237
24	6	0	3.695529	-1.436733	0.134965
25	1	0	2.689708	-2.648153	1.604809
26	1	0	4.416369	-0.057608	-1.352965
27	1	0	4.638946	-1.966532	0.221613

3a

Standard orientation:

Center Number	Atomic Number	Atomic Type	Coordinates (Angstroms) X	Y	Z
1	6	0	-0.051902	0.839048	0.193718
2	6	0	-1.267623	-0.048574	0.058884
3	6	0	-1.127921	-1.435463	-0.029900
4	6	0	-2.557762	0.496316	0.057184
5	6	0	-2.250958	-2.257961	-0.109042
6	1	0	-0.138791	-1.880812	-0.034660
7	6	0	-3.674915	-0.324726	-0.017056
8	1	0	-2.682909	1.575157	0.086408
9	6	0	-3.527029	-1.708779	-0.100288
10	1	0	-2.119897	-3.333501	-0.178443
11	1	0	-4.666082	0.118481	-0.019201
12	1	0	-4.400901	-2.349618	-0.163245
13	6	0	-0.132661	1.981664	1.146682
14	1	0	0.767666	2.240618	1.697711

15	1	0	-1.063794	2.114980	1.691949
16	16	0	-0.147054	2.503192	-0.584687
17	6	0	1.280512	0.150142	0.077752
18	6	0	1.855732	-0.096142	-1.169506
19	6	0	1.920704	-0.314631	1.227201
20	6	0	3.057683	-0.788614	-1.265871
21	1	0	1.356867	0.270059	-2.062651
22	6	0	3.126136	-1.006949	1.132970
23	1	0	1.466465	-0.140024	2.199450
24	6	0	3.696601	-1.244716	-0.113841
25	1	0	3.499935	-0.968607	-2.240802
26	1	0	3.617678	-1.359867	2.034310
27	1	0	4.637044	-1.781636	-0.189167

17

Standard orientation:

Center Number	Atomic Number	Atomic Type	Coordinates (Angstroms) X	Y	Z
1	6	0	2.214407	0.195185	-0.329859
2	6	0	-2.214407	-0.195185	-0.329859
3	6	0	1.749556	1.289232	-1.205353
4	6	0	2.453215	1.693430	-2.350128
5	6	0	0.552979	1.957628	-0.896985
6	6	0	1.961016	2.702935	-3.171329
7	1	0	3.405548	1.226851	-2.584826
8	6	0	0.075410	2.981010	-1.704835
9	1	0	-0.001255	1.650243	-0.015538
10	6	0	0.773418	3.355039	-2.852681
11	1	0	2.523895	2.996065	-4.052407
12	1	0	-0.855707	3.474771	-1.440143
13	1	0	0.399364	4.152792	-3.486539
14	6	0	-1.749556	-1.289232	-1.205353
15	6	0	-2.453215	-1.693430	-2.350128
16	6	0	-0.552979	-1.957628	-0.896985
17	6	0	-1.961016	-2.702935	-3.171329
18	1	0	-3.405548	-1.226851	-2.584826
19	6	0	-0.075410	-2.981010	-1.704835
20	1	0	0.001255	-1.650243	-0.015538
21	6	0	-0.773418	-3.355039	-2.852681
22	1	0	-2.523895	-2.996065	-4.052407
23	1	0	0.855707	-3.474771	-1.440143
24	1	0	-0.399364	-4.152792	-3.486539
25	6	0	3.563716	-2.260823	-0.230791
26	1	0	4.010270	-3.063125	-0.800435
27	1	0	3.615954	-2.245480	0.847703
28	6	0	-3.563716	2.260823	-0.230791
29	1	0	-3.615954	2.245480	0.847703
30	1	0	-4.010270	3.063125	-0.800435
31	16	0	2.896360	-1.077817	-1.142111
32	16	0	-2.896360	1.077817	-1.142111
33	6	0	2.046715	0.276255	1.128642
34	6	0	2.200536	1.516789	1.772601
35	6	0	1.719275	-0.837566	1.919374
36	6	0	2.057255	1.630455	3.149345
37	1	0	2.444739	2.391554	1.177307

38	6	0	1.571762	-0.719619	3.296691
39	1	0	1.567251	-1.806166	1.452335
40	6	0	1.749556	0.511430	3.921070
41	1	0	2.194766	2.597955	3.623229
42	1	0	1.301522	-1.594011	3.880882
43	1	0	1.639272	0.600505	4.997638
44	6	0	-2.046715	-0.276255	1.128642
45	6	0	-2.200536	-1.516789	1.772601
46	6	0	-1.719275	0.837566	1.919374
47	6	0	-2.057255	-1.630455	3.149345
48	1	0	-2.444739	-2.391554	1.177307
49	6	0	-1.571762	0.719619	3.296691
50	1	0	-1.567251	1.806166	1.452335
51	6	0	-1.749556	-0.511430	3.921070
52	1	0	-2.194766	-2.597955	3.623229
53	1	0	-1.301522	1.594011	3.880882
54	1	0	-1.639272	-0.600505	4.997638

TS7

Standard orientation:

Center Number	Atomic Number	Atomic Type	Coordinates (Angstroms) X	Y	Z
1	6	0	-2.111014	-1.179490	0.310352
2	6	0	1.846281	0.773376	0.776769
3	6	0	-2.929681	-0.493247	-0.695584
4	6	0	-2.976288	-0.986272	-2.013399
5	6	0	-3.689172	0.655909	-0.404658
6	6	0	-3.743852	-0.360201	-2.986435
7	1	0	-2.406645	-1.875874	-2.263462
8	6	0	-4.470617	1.266832	-1.376576
9	1	0	-3.656351	1.081981	0.594361
10	6	0	-4.500990	0.768049	-2.676825
11	1	0	-3.758520	-0.764950	-3.994101
12	1	0	-5.044577	2.152418	-1.119584
13	1	0	-5.101429	1.254534	-3.438658
14	6	0	2.821307	0.448464	-0.265417
15	6	0	3.885228	-0.454225	-0.070335
16	6	0	2.678516	1.014384	-1.548295
17	6	0	4.726326	-0.809203	-1.115890
18	1	0	4.057438	-0.884750	0.911521
19	6	0	3.535795	0.669786	-2.585519
20	1	0	1.864923	1.708287	-1.734261
21	6	0	4.559600	-0.254312	-2.383963
22	1	0	5.531228	-1.515228	-0.933366
23	1	0	3.391478	1.117275	-3.564564
24	1	0	5.224172	-0.528093	-3.196940
25	6	0	-1.453639	-1.462033	3.007154
26	1	0	-1.796351	-1.392598	4.032068
27	1	0	-0.675959	-2.167108	2.760994
28	6	0	0.569107	0.096811	3.173330
29	1	0	0.006495	1.020127	3.126954
30	1	0	0.693666	-0.408645	4.121068
31	16	0	-2.594542	-0.987892	1.903374
32	16	0	1.740635	-0.282389	2.068724
33	6	0	-0.903099	-1.927462	-0.054001

34	6	0	0.014528	-1.382314	-0.969468
35	6	0	-0.610146	-3.183015	0.508187
36	6	0	1.183182	-2.063628	-1.298806
37	1	0	-0.192324	-0.411087	-1.413674
38	6	0	0.567216	-3.848255	0.197491
39	1	0	-1.328090	-3.641317	1.183804
40	6	0	1.475366	-3.285978	-0.701920
41	1	0	1.883914	-1.621690	-2.001690
42	1	0	0.771562	-4.816581	0.644945
43	1	0	2.399302	-3.802043	-0.943861
44	6	0	1.019035	1.988962	0.682022
45	6	0	-0.379744	1.936619	0.736092
46	6	0	1.638130	3.236310	0.499438
47	6	0	-1.137592	3.099374	0.614816
48	1	0	-0.871300	0.972691	0.843472
49	6	0	0.880558	4.394539	0.386589
50	1	0	2.722411	3.281342	0.446445
51	6	0	-0.511696	4.330336	0.445413
52	1	0	-2.221140	3.038304	0.636050
53	1	0	1.376414	5.351181	0.252415
54	1	0	-1.103649	5.235616	0.353137

18

Standard orientation:

Center Number	Atomic Number	Atomic Type	Coordinates (Angstroms) X	Y	Z
1	6	0	0.660278	2.685283	-0.449920
2	6	0	-0.660278	-2.685283	-0.449920
3	6	0	0.203003	3.972828	-0.950072
4	6	0	0.181674	5.107515	-0.107840
5	6	0	-0.203003	4.148801	-2.290135
6	6	0	-0.240438	6.341562	-0.578953
7	1	0	0.527733	5.011923	0.916267
8	6	0	-0.626436	5.386184	-2.753628
9	1	0	-0.194801	3.294971	-2.959148
10	6	0	-0.651228	6.490626	-1.903502
11	1	0	-0.236485	7.197825	0.088767
12	1	0	-0.944824	5.488449	-3.786653
13	1	0	-0.981015	7.457596	-2.269761
14	6	0	-0.203003	-3.972828	-0.950072
15	6	0	-0.181674	-5.107515	-0.107840
16	6	0	0.203003	-4.148801	-2.290135
17	6	0	0.240438	-6.341562	-0.578953
18	1	0	-0.527733	-5.011923	0.916267
19	6	0	0.626436	-5.386184	-2.753628
20	1	0	0.194801	-3.294971	-2.959148
21	6	0	0.651228	-6.490626	-1.903502
22	1	0	0.236485	-7.197825	0.088767
23	1	0	0.944824	-5.488449	-3.786653
24	1	0	0.981015	-7.457596	-2.269761
25	6	0	0.761149	-0.010926	-1.264179
26	1	0	1.141551	-0.630370	-2.082214
27	1	0	1.135638	-0.421047	-0.322392
28	6	0	-0.761149	0.010926	-1.264179
29	1	0	-1.135638	0.421047	-0.322392

30	1	0	-1.141551	0.630370	-2.082214
31	16	0	1.484139	1.639115	-1.590404
32	16	0	-1.484139	-1.639115	-1.590404
33	6	0	-0.558336	-2.308236	0.951721
34	6	0	0.525610	-2.755124	1.738297
35	6	0	-1.516463	-1.476987	1.567960
36	6	0	0.615630	-2.427218	3.083734
37	1	0	1.297979	-3.362107	1.275630
38	6	0	-1.419399	-1.151653	2.912925
39	1	0	-2.364471	-1.129932	0.984658
40	6	0	-0.361115	-1.633105	3.682190
41	1	0	1.456666	-2.789524	3.667671
42	1	0	-2.176754	-0.519481	3.366478
43	1	0	-0.290984	-1.379237	4.735562
44	6	0	0.558336	2.308236	0.951721
45	6	0	1.516463	1.476987	1.567960
46	6	0	-0.525610	2.755124	1.738297
47	6	0	1.419399	1.151653	2.912925
48	1	0	2.364471	1.129932	0.984658
49	6	0	-0.615630	2.427218	3.083734
50	1	0	-1.297979	3.362107	1.275630
51	6	0	0.361115	1.633105	3.682190
52	1	0	2.176754	0.519481	3.366478
53	1	0	-1.456666	2.789524	3.667671
54	1	0	0.290984	1.379237	4.735562

TS8

Standard orientation:

Center Number	Atomic Number	Atomic Type	Coordinates (Angstroms) X	Y	Z
1	6	0	1.245073	-0.544649	-0.402548
2	6	0	-1.245073	0.544649	-0.402548
3	6	0	-0.714794	0.260474	-3.141417
4	16	0	-1.796326	-0.373110	-1.817124
5	6	0	0.714794	-0.260474	-3.141417
6	16	0	1.796326	0.373110	-1.817124
7	1	0	-1.208002	-0.081493	-4.057569
8	1	0	-0.733656	1.352860	-3.137931
9	1	0	0.733656	-1.352860	-3.137931
10	1	0	1.208002	0.081493	-4.057569
11	6	0	1.762289	-0.091498	0.891065
12	6	0	1.392010	-0.734681	2.090390
13	6	0	2.667098	0.989292	0.995939
14	6	0	1.869977	-0.298909	3.319922
15	1	0	0.717413	-1.581731	2.063650
16	6	0	3.145318	1.415993	2.226471
17	1	0	3.000293	1.505118	0.100378
18	6	0	2.745242	0.780313	3.401305
19	1	0	1.549030	-0.811083	4.222071
20	1	0	3.842894	2.247249	2.266674
21	1	0	3.118334	1.116926	4.363276
22	6	0	-1.762289	0.091498	0.891065
23	6	0	-1.392010	0.734681	2.090390
24	6	0	-2.667098	-0.989292	0.995939
25	6	0	-1.869977	0.298909	3.319922

26	1	0	-0.717413	1.581731	2.063650
27	6	0	-3.145318	-1.415993	2.226471
28	1	0	-3.000293	-1.505118	0.100378
29	6	0	-2.745242	-0.780313	3.401305
30	1	0	-1.549030	0.811083	4.222071
31	1	0	-3.842894	-2.247249	2.266674
32	1	0	-3.118334	-1.116926	4.363276
33	6	0	-1.033399	2.006725	-0.612316
34	6	0	0.000826	2.719826	0.003982
35	6	0	-1.930931	2.726980	-1.420499
36	6	0	0.137292	4.093231	-0.177614
37	1	0	0.714244	2.195243	0.624506
38	6	0	-1.796326	4.098212	-1.600338
39	1	0	-2.751411	2.198975	-1.898007
40	6	0	-0.757731	4.790535	-0.981088
41	1	0	0.954602	4.614524	0.311871
42	1	0	-2.512017	4.628636	-2.221335
43	1	0	-0.650853	5.861457	-1.123120
44	6	0	1.033399	-2.006725	-0.612316
45	6	0	-0.000826	-2.719826	0.003982
46	6	0	1.930931	-2.726980	-1.420499
47	6	0	-0.137292	-4.093231	-0.177614
48	1	0	-0.714244	-2.195243	0.624506
49	6	0	1.796326	-4.098212	-1.600338
50	1	0	2.751411	-2.198975	-1.898007
51	6	0	0.757731	-4.790535	-0.981088
52	1	0	-0.954602	-4.614524	0.311871
53	1	0	2.512017	-4.628636	-2.221335
54	1	0	0.650853	-5.861457	-1.123120

5a

Standard orientation:

Center Number	Atomic Number	Atomic Type	Coordinates (Angstroms) X	Y	Z
1	6	0	0.634256	-0.441648	-0.506045
2	6	0	-0.634263	-0.441639	0.506049
3	6	0	-0.560173	-3.274564	0.512851
4	16	0	-0.433147	-1.888787	1.672302
5	6	0	0.560120	-3.274572	-0.512846
6	16	0	0.433117	-1.888794	-1.672297
7	1	0	-0.498251	-4.165473	1.146600
8	1	0	-1.540441	-3.261106	0.025674
9	1	0	1.540388	-3.261132	-0.025670
10	1	0	0.498181	-4.165480	-1.146596
11	6	0	0.770503	0.839427	-1.369670
12	6	0	1.250291	2.007657	-0.758351
13	6	0	0.548000	0.880738	-2.749079
14	6	0	1.432434	3.179844	-1.480164
15	1	0	1.492585	1.997332	0.299267
16	6	0	0.739497	2.053177	-3.479576
17	1	0	0.227958	-0.006889	-3.285703
18	6	0	1.170905	3.212084	-2.848052
19	1	0	1.793358	4.067636	-0.970184
20	1	0	0.554432	2.047930	-4.549255
21	1	0	1.319401	4.125408	-3.415619

22	6	0	-0.770490	0.839439	1.369672
23	6	0	-1.250247	2.007679	0.758347
24	6	0	-0.548001	0.880748	2.749084
25	6	0	-1.432373	3.179870	1.480156
26	1	0	-1.492530	1.997359	-0.299274
27	6	0	-0.739480	2.053192	3.479576
28	1	0	-0.227983	-0.006884	3.285714
29	6	0	-1.170858	3.212107	2.848047
30	1	0	-1.793272	4.067669	0.970169
31	1	0	-0.554426	2.047943	4.549258
32	1	0	-1.319341	4.125436	3.415610
33	6	0	2.035982	-0.578513	0.155679
34	6	0	2.320051	-0.297287	1.492606
35	6	0	3.123292	-0.878468	-0.680050
36	6	0	3.624836	-0.344153	1.983437
37	1	0	1.532535	-0.028485	2.177006
38	6	0	4.423092	-0.933302	-0.193623
39	1	0	2.946029	-1.066759	-1.733304
40	6	0	4.684320	-0.671550	1.148906
41	1	0	3.801317	-0.122237	3.031580
42	1	0	5.235118	-1.174629	-0.872842
43	1	0	5.698667	-0.713823	1.533313
44	6	0	-2.035990	-0.578487	-0.155678
45	6	0	-2.320055	-0.297255	-1.492604
46	6	0	-3.123304	-0.878440	0.680048
47	6	0	-3.624839	-0.344111	-1.983438
48	1	0	-1.532536	-0.028458	-2.177002
49	6	0	-4.423103	-0.933265	0.193618
50	1	0	-2.946042	-1.066739	1.733301
51	6	0	-4.684327	-0.671505	-1.148910
52	1	0	-3.801317	-0.122189	-3.031580
53	1	0	-5.235132	-1.174591	0.872835
54	1	0	-5.698674	-0.713769	-1.533319
